# Diaphragm position can be accurately estimated from the scattering of a parallel transmit RF coil at 7 T

**DOI:** 10.1002/mrm.26866

**Published:** 2017-08-03

**Authors:** Aaron T. Hess, Elizabeth M Tunnicliffe, Christopher T. Rodgers, Matthew D. Robson

**Affiliations:** ^1^ University of Oxford Centre for Clinical Magnetic Resonance Research, Division of Cardiovascular Medicine, Radcliffe Department of Medicine Oxford United Kingdom

**Keywords:** motion, respiration, navigator, RF scattering, parallel transmit, free breathing

## Abstract

**Purpose:**

To evaluate the use of radiofrequency scattering of a parallel transmit coil to track diaphragm motion.

**Methods:**

Measurements made during radiofrequency excitation on an 8‐channel parallel transmit coil by the directional couplers of the radiofrequency safety monitor were combined and converted into diaphragm position. A 30‐s subject‐specific calibration with an MRI navigator was used to determine a diaphragm estimate from each directional‐coupler measure. Seven healthy volunteers were scanned at 7 T, in which images of the diaphragm were continuously acquired and directional couplers were monitored during excitation radiofrequency pulses. The ability to detect coughing was evaluated in one subject. The method was implemented on the scanner and evaluated for diaphragm gating of a free‐breathing cardiac cine.

**Results:**

Six of the seven scans were successful. In these subjects, the root mean square difference between MRI and scattering estimation of the superior–inferior diaphragm position was 1.4 ± 0.5 mm. On the scanner, the position was calculated less than 2 ms after every radiofrequency pulse. A prospectively gated (echocardiogram and respiration) high‐resolution free‐breathing cine showed no respiratory artifact and sharp blood‐myocardium definition.

**Conclusions:**

Transmit coil scattering is sensitive to diaphragm motion and provides rapid, quantitative, and accurate monitoring of respiration. Magn Reson Med 79:2164–2169, 2018. © 2017 The Authors Magnetic Resonance in Medicine published by Wiley Periodicals, Inc. on behalf of International Society for Magnetic Resonance in Medicine. This is an open access article under the terms of the Creative Commons Attribution License, which permits use, distribution and reproduction in any medium, provided the original work is properly cited.

## INTRODUCTION

Motion has the potential to severely corrupt MR images of the abdomen and thorax, primarily due to the superior–inferior motion of the diaphragm during the respiratory cycle. A number of solutions exist to address this, which can broadly be categorized into breath‐hold methods 1, 2 and respiratory gating or binning methods 3, 4, 5. Breath‐holding remains the mainstay when acquisition durations can be restricted to fewer than 15 s. The alternative is to resolve the respiratory cycle, which comes with a trade‐off between measuring the diaphragm position by introducing a sequence design constraint, such as a navigator echo 3, 6, encoding strategies with self‐gating attributes 4, or the use of a bellows that provides a surrogate measure of diaphragm location 5, 7, 8.

Radiofrequency (RF) coils in MRI are sensitive to changes in the conductivity of tissues within their electric fields. In receiver arrays this effect has been monitored by measuring noise characteristics 9, and by introducing a reference transmitter (termed a “pilot‐tone navigator”) 10. Use of the transmit RF coil for monitoring dates back to 1988 when it was reported by Buikman et al. 11, who used a directional coupler (DICO) to monitor the returned power from the coil. More recently, pickup coils on a 3T parallel transmit (pTx) body coil were used to observe changes in the transmit coil current with respiration in multiple transmitters 12; in later work, the authors identified that drifts in the transmit amplifiers confounded the continuous monitoring of respiration 13, and the use of low‐power monitoring pulses was proposed. Like bellows, these methods provide a subject‐dependent surrogate measure of respiration.

At 7 T, abdominal and thoracic imaging uses local‐transmit pTx 14, 15 to improve homogeneity of the $B_{1}^{+}$ fields and reduce the total specific absorption rate burden. An intrinsic part of the safety monitoring in many MRI systems is the use of DICOs to measure the transmit current in each channel during RF pulses. Each channel of the transmit array is strongly coupled to the underlying tissue. Thus, we hypothesize that these DICO measurements, which are already collected by the system during the RF pulses in any standard sequence, in conjunction with a calibration process, contain sufficient respiratory information to enable accurate tracking of the diaphragm.

## THEORY

A pTx RF coil can be characterized as an *N*‐port electrical network, in which *N* is the number of transmit channels. This network has *N* inputs (the voltage and current into each channel) and *N* outputs (the voltage and current returned from each channel). During RF excitation, DICOs of a pTx system measure a time series of return and forward voltages ( ${\vec{v}}_{ret}\left(t\right)$ and ${\vec{v}}_{fwd}\left(t\right)$, respectively), both complex vectors of length *N*. The voltages ${\vec{v}}_{ret}\left(t\right)$ and ${\vec{v}}_{fwd}\left(t\right)$ are related to each other by the time‐dependent scattering matrix, S(t), assuming no measurement error, by

(1) $${\vec{v}}_{ret}\left(t\right)=S\left(t\right){\vec{v}}_{fwd}\left(t\right)$$

Each element of the *N* × *N* matrix S(t) is a ratio of input to output voltages of the network. The load of this network is characterized by the impedance matrix Z(t). The impedance $Z_{i,j}\left(t\right)$ is the ratio of induced voltage on port i to a feed current on port j, as such S(t) and Z(t) are related using the characteristic impedance $Z_{0}$ (usually 50 Ω) using the following equation, in which I is the identity matrix 16:

(2) $$S\left(t\right)=\left(\frac{1}{Z_{0}}Z\left(t\right)-I\right){\left(\frac{1}{Z_{0}}Z\left(t\right)+I\right)}^{-1}$$

Following the notation of Malmivuo and Plonsey 17, the induced voltage is proportional to the dot product of the lead fields ( ${\vec{J}}_{Li}\left(\vec{x},t\right)$) of channels i and j, in which the lead field, ${\vec{J}}_{Li}\left(\vec{x},t\right)$, is the current density at the spatial location $\vec{x}$ per unit input current on channel i 17 in units of m^−2^. Equation (3) is used to quantify the sensitivity of the network to the underlying tissue complex conductivity ( $\sigma \left(\vec{x},t\right)$, in units of Sm^−1^) for each element to produce the symmetric matrix Z(t).

(3) $$Z_{i,j}\left(t\right)=\overset{}{\underset{V}{\int }}\frac{1}{\sigma \left(\vec{x},t\right)}{\vec{J}}_{Li}\left(\vec{x},t\right)\cdot {\vec{J}}_{Lj}\left(\vec{x},t\right)\;dv$$

Multiple tissue types, each with different $\sigma \left(\vec{x},t\right)$, are present within each coil element's region of sensitivity, ${\vec{J}}_{Li}\left(\vec{x},t\right)$. As the subject breathes, the tissue changes, along with the complex conductivity of the lung tissue itself 18, 19. These physiological perturbations in Z(t) give rise to similar changes in S(t).

However, during a pTx MRI scan that uses static $B_{1}^{+}$ shimming (i.e., all channels transmit the same pulse), a constant relationship is present among all ports, and Equation (1) cannot be inverted to determine S(t). Thus, ${\vec{v}}_{ret}\left(t\right)$ can be used as a measure of S(t), but ${\vec{v}}_{ret}\left(t\right)$ is also perturbed by any instability in any of the *N* RF amplifiers. Although these instabilities will be within the amplifier specification, they can be greater than the physiological effects. Previously, it has been suggested that ${\vec{v}}_{ret}\left(t\right)$ can be normalized on a channel‐by‐channel basis using ${\vec{v}}_{fwd}\left(t\right)$ 13, 20. However, this does not take into account the presence of off‐diagonal terms in S(t).

## METHODS

All experiments were carried out on a Magnetom 7 T scanner (Siemens, Erlangen, Germany) equipped with eight pTx channels and local specific absorption rate monitoring (software version VB17, step 2.3). The local specific absorption rate monitor consists of eight directional couplers, one for each transmit channel, which monitor the complex forward ${\vec{v}}_{fwd}\left(t\right)$ and returned ${\vec{v}}_{ret}\left(t\right)$ RF voltage on each transmit channel. They are demodulated by the carrier frequency and connected to the MR receivers' analog‐to‐digital converter that digitizes them at 1 MHz during all RF transmissions. The digitized voltages are delivered in real time to the specific absorption rate monitor. An eight‐channel local transmit‐receive RF coil was custom‐built using the transmission line method, consisting of four elements positioned posteriorly and four anteriorly. Each element is 150 mm long and separated by 50‐mm center to center and positioned for maximum coverage of the heart 15, 21, 22, 23, 24.

### Magnitude and Sensitivity of Respiratory Motion in Scattering

In one healthy volunteer S(t) was measured by transmitting frequency‐multiplexed, 5 ms, Gaussian RF pulses every 10 ms for 20 s during free breathing. The frequency spacing between each channel was 2 kHz. The scattering matrix, S, was calculated for each RF pulse using the DICO‐measured voltages for each channel *i*, ${\vec{v}}_{fwd,i}$, and ${\vec{v}}_{ret,i}$. A Fourier transform was used to separate out the frequency components, *j*, corresponding to each channel, forming the matrix $V_{i,j}^{fwd}$ and $V_{i,j}^{ret}$. $V_{}^{fwd}$ is a diagonal matrix, and we define $\;{\vec{V}}^{fwd}=\text{diag}\left(V_{}^{fwd}\right)$. Using Equation (1), S was calculated for each RF pulse, which were concatenated to form the time‐varying matrix S(t), with 10‐ms time resolution.

To evaluate the magnitude of amplifier noise and drift on DICO measurements made during a static $B_{1}^{+}$ shim, two synthetic return vectors were formed using this S(t). The amplifier effects were removed by taking the average over all time points of ${\vec{V}}^{fwd}\left(t\right)$ to form ${\vec{V}}_{}^{ret,ideal}\left(t\right)=S\left(t\right)\bar{{\vec{V}}^{fwd}}$. A second return vector was formed, preserving amplifier effects, as ${\vec{V}}_{}^{ret,measured}\left(t\right)=\;S\left(t\right){\vec{V}}^{fwd}\left(t\right)$.

Electromagnetic simulations of the transmission line coil on a model human thorax were carried out to understand the spatial sensitivity profiles of S and are included in the supplementary information.

### Algorithm

S(t) can be modeled as the sum of a temporally invariant matrix $S_{0}$ and a motion‐dependent matrix $\Delta S_{motion}\left(t\right)$ as follows:

(4) $$S\left(t\right)=S_{0}+\Delta S_{motion}\left(t\right)$$

$S_{0}$ is measured before the target image acquisition using the frequency multiplexed RF pulse, as done previously, at an arbitrary respiratory state. During the $B_{1}^{+}$ shimmed target image acquisition, all channels transmit with identical frequency. To track respiratory motion, a vector $\vec{\Gamma }\left(t\right)$ is calculated from an element‐wise division of N × 1 vector ${\vec{v}}_{ret}\left(t\right)$ by the return expected given $S_{0}$ (expected return = $S_{0}{\vec{v}}_{fwd}\left(t\right)$) as follows:

(5) $$\vec{\Gamma }\left(t\right)=\frac{{\vec{v}}_{ret}\left(t\right)}{\left[S_{0}{\vec{v}}_{fwd}\left(t\right)\right]}=1+\frac{\left[\Delta S_{motion}\left(t\right){\vec{v}}_{fwd}\left(t\right)\right]}{\left[S_{0}{\vec{v}}_{fwd}\left(t\right)\right]}$$

The complex vector $\vec{\Gamma }\left(t\right)$ is determined from the central 50 µs of DICO data sampled for each RF pulse. A diaphragm position estimate ( $d_{}^{est}\left(t\right)$) is formed by summing the real and imaginary components of $\vec{\Gamma }\left(t\right)$ with weighting vectors ${\vec{m}}_{r}$ and ${\vec{m}}_{i}$ as follows:

(6) $$d_{}^{est}\left(t\right)=real\left(\vec{\Gamma }\left(t\right)\right)\cdot {\vec{m}}_{r}+imag\left(\vec{\Gamma }\left(t\right)\right)\cdot {\vec{m}}_{i}+c$$

where c is a constant offset.

${\vec{m}}_{r}$, ${\vec{m}}_{i}$, and c are determined in a calibration in which the diaphragm position is measured with MRI and $\vec{\Gamma }\left(t\right)$ is recorded for each diaphragm image and determined using a least‐squares optimization.

### Validation

Seven healthy male volunteers were recruited according to our institution's ethical practices. A $B_{1}^{+}$ shim was applied that adjusts only the phase of each channel to maximize the minimum $B_{1}^{+}$ over the edge of the diaphragm for both inspiration and expiration 25. A sagittal spoiled gradient echo (SPGR) image was prescribed through the right hemidiaphragm, from which both a diaphragm position and $\vec{\Gamma }\left(t\right)$ can be determined. The SPGR had a flip angle between 2° and 10°, a train of 71 sinc RF pulses of 1.0 ms long, repetition time (TR)/echo time of 5.0/2.0 ms, generalized autocalibrating partial parallel acquisition iPAT factor of 2 and 6:8 phase partial Fourier, matrix size of 304 × 156, and field of view of 300 × 300 mm^2^ for a total acquisition duration of 322 ms. This was repeated for 2 min 45 s, giving a total of 512 images and 512 × 71 sets of forward and returned voltage vectors.

The diaphragm images along with digitized forward and returned RF voltages were processed in MATLAB (MathWorks, Natick, MA, USA) and $\vec{\Gamma }\left(t\right)$ determined retrospectively. The diaphragm position in the images was determined using edge detection and upsampled to the RF pulse rate using linear interpolation. The first 93 images (30 s) were used for calibration; the remaining 420 images were used to evaluate the estimate $d_{}^{est}\left(t\right)$. No temporal filtering was applied to the diaphragm estimate.

A frequency analysis on the difference between $d_{}^{est}\left(t\right)$ and diaphragm position in the target images for each subject was done with a Fourier transform of the difference, and an energy‐conserving Hanning apodization of 0.15 Hz.

One subject was instructed to cough four times during the acquisition after the initial 30‐s calibration, to evaluate the response of the system to nonperiodic respiration. For this assessment, a Kalman filter 26 was used to estimate diaphragm velocity from both the scattering and image‐based diaphragm positions. The Kalman filter gain ( $\Delta^{*}$) was 2.9 s^−1^ for velocity and 0.16 for position 26. Velocities out of the standard range ( −25 to 25 mm/s) are taken to be the start of coughing.

### Real‐Time Pulse Sequence

To evaluate the practicality of this method for prospective, real‐time, respiratory‐state detection, a pulse sequence was developed. This contained two parts, the first being calibration using an SPGR diaphragm navigator 27 sequence with three imaging RF pulses between each navigator and lasting 30 s, and the second part being the target imaging acquisition. The navigator had a matrix size of 210 × 21, field of view of 300 × 300 mm, slice thickness of 13 mm, TR/echo time 3.5/1.5 ms. The three target image RF pulses were used to determine $\vec{\Gamma }$ during calibration. The target image acquisition part of the sequence received feedback on the diaphragm position each TR. The sequence opened or closed a respiratory gate for each TR, as determined by the diaphragm position estimated. The time for this feedback loop was 35 ms from measurement to an adapted execution. This included a 2‐ms calculation time, 15‐ms pulse pre‐preparation time, and an 18‐ms buffer for fluctuations in network communication and computation time.

This algorithm was implemented on the scanner's image reconstruction system. It consisted of an image reconstruction and edge detection for the navigator images, a functional unit to perform the calibration, and online calculation of the diaphragm position from scattering, which is transmitted back to the MPCUs of the pTx.

For this work the target imaging sequence was a 2D cine SPGR pulse sequence. A horizontal long‐axis cine of the right side of the heart was acquired in a single healthy volunteer as follows: separate $B_{1}^{+}$ shims were set for the right hemidiaphragm and right side of the heart for the navigator and image, respectively, with a ± 3‐mm acceptance window at end expiration. The cine had a flip angle of approximately 10°, TR/echo time 5.0/2.0 ms, matrix 304 × 256, field of view 300 × 253 mm, 29 ms per cardiac phase, and slice thickness of 3 mm.

## RESULTS

### Magnitude and Sensitivity of Respiratory Motion in Scattering

**Figure 1:**
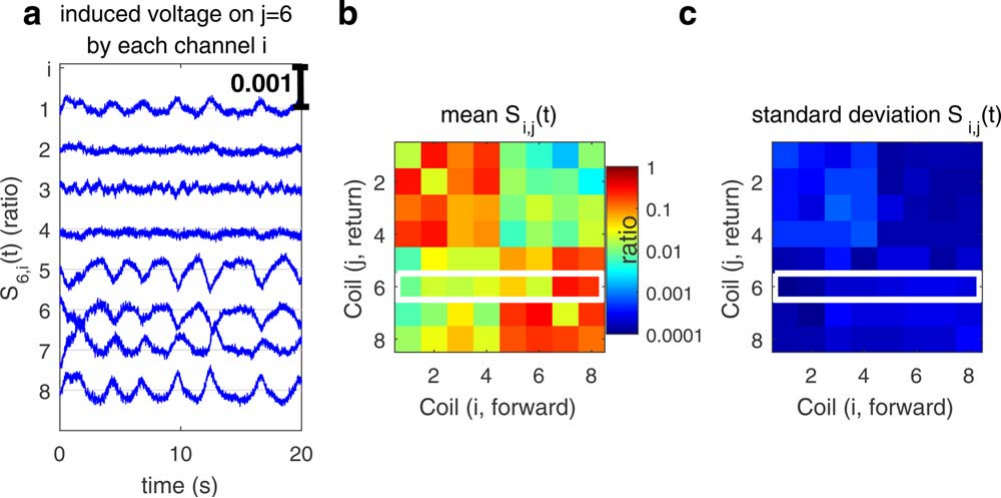
The scattering matrix. **a**: A recording of the absolute change in scattering with time for all components returned to channel 6, the sixth row of the scattering matrix ***S*** *(t)*, the scale bar is 1:1000. **b**: Temporal average scattering matrix. **c**: The temporal standard deviation. Note the mean scattering matrix can be more than 100 times larger than the temporal fluctuations, and fluctuations occur with a similar magnitude on the diagonal and off‐diagonal elements. Note also the larger intracoil scattering for the anterior 1, 2, 3, 4 and posterior 5, 6, 7, 8 sets of coils. See also Supporting Figures S1 and S2 for spatial distribution of the sensitivity of these measures.

**Figure 2:**
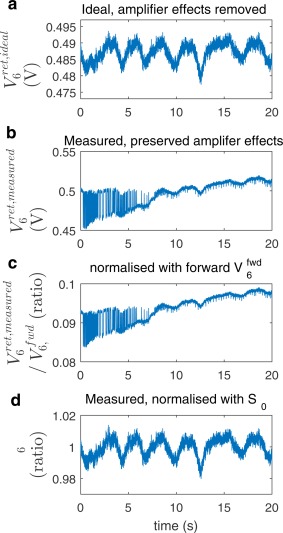
Formation of $\Gamma_{6}\left(t\right)$ showing element 6 and the data that are plotted in Figure 1. **a**: Plots ${\vec{V}}_{}^{ret,ideal}\left(t\right)$, element 6 of the product of S(t) and a constant vector to remove all amplifier effects. **b**: ${\vec{V}}_{}^{ret,measured}\left(t\right)$, which includes amplifier drift and amplifier noise. **c**: Plot of the effect of normalizing ${\vec{V}}_{}^{ret,measured}\left(t\right)$ using $v_{6}^{fwd}\left(t\right)$. **d**: $\Gamma_{6}\left(t\right)$, which is the return normalized using **S**_**0**_.

Figure 1 shows the sixth row of the scattering matrix, measured continuously for 20 s, its mean, and its standard deviation. These data show that the physiological motion exceeds the DICO measurement error, that $\Delta S_{motion}\left(t\right)$ has similar magnitude both on and off the diagonal. Figure 2 plots the components of the synthetic return vectors for channel 6. This figure demonstrates that the amplifier influences on $V_{6}^{ret,measured}\left(t\right)$ exceed the physiological fluctuations, which also holds in the other channels. Figure 2 shows that normalization of $V_{6}^{ret,measured}\left(t\right)$ using the forward voltage of channel 6 is insufficient to recover the respiratory information, which is recovered with $\Gamma_{6}\left(t\right)$. See also Supporting Figures S1 and S2 for the sensitivity profiles of the impedance matrix Z.

### Validation

In six subjects, the root mean square error in $d_{}^{est}\left(t\right)$ (difference compared with MRI) was 1.4 ± 0.5 mm, but in one subject there was a gradual increase in $d_{}^{est}\left(t\right)$ to 20 mm in diaphragm position by the end of the scan. On inspection in this subject, the elements of $\vec{\Gamma }\left(t\right)$ ranged from 0.6 to 1.4, substantially out of its expected range of 0.95 to 1.05. This subject was excluded from further analysis, as this implies a large change in $S_{0}$ between its measurement and the start of the validation scan.

**Figure 3:**
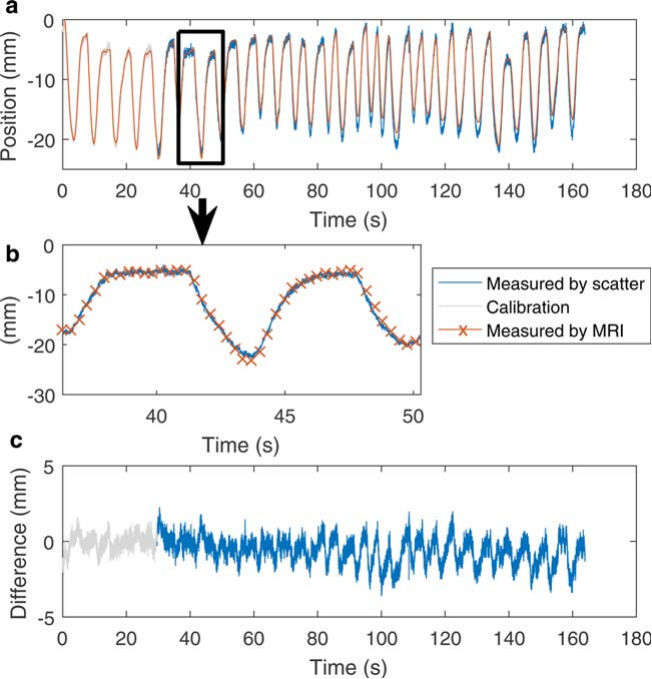
**a**: Plot of estimated and measured diaphragm position. **b**: Extract from (**a**) showing the temporal resolution of the estimate compared with imaging. **c**: Difference between the estimate and MRI at the RF pulse‐sampling frequency.

Figure 3 compares the scattering‐estimated diaphragm position with the MRI‐measured position for one of the successful subjects. The difference between the two diaphragm positions show a small but systematic variation as a function of respiration. The frequency analysis from one of the six typical subjects is shown in the Supporting Figure S3. This shows three frequency bands: a respiratory band with an amplitude of 1.0 ± 0.3 mm, a cardiac band with an amplitude of 0.4 ± 0.1 mm, and a band at the imaging frequency (3.1 Hz) with an amplitude of 0.1 ± 0.1 mm.

**Figure 4:**
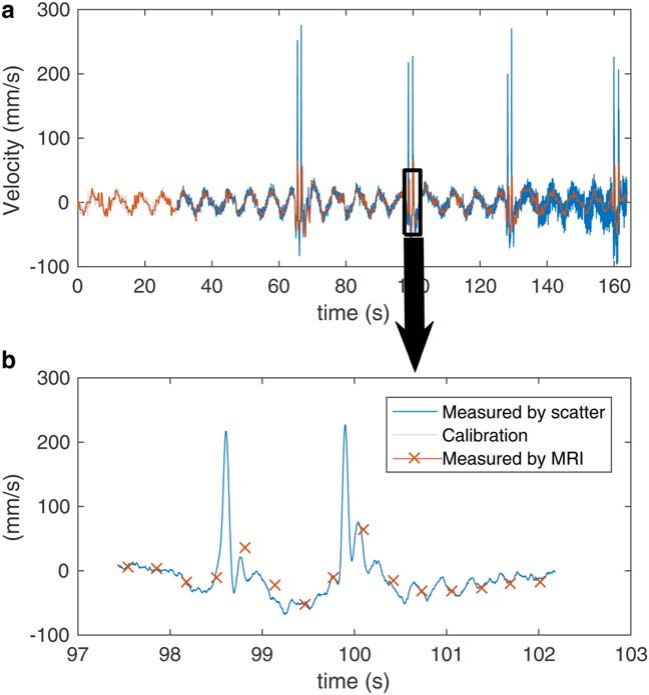
Diaphragm velocity estimated from the diaphragm position estimates and MRI with an excerpt showing the fast response time of the scattering‐based estimate when observing coughing (140 ms faster than the MRI measure).

During the scan with deliberate coughing, the velocity estimated using both methods exceeded the standard range of ± 25 mm/s for all four coughs. This plot of Kalman‐filtered velocity is shown in Figure 4.

### Real‐Time Pulse Sequence

**Figure 5:**
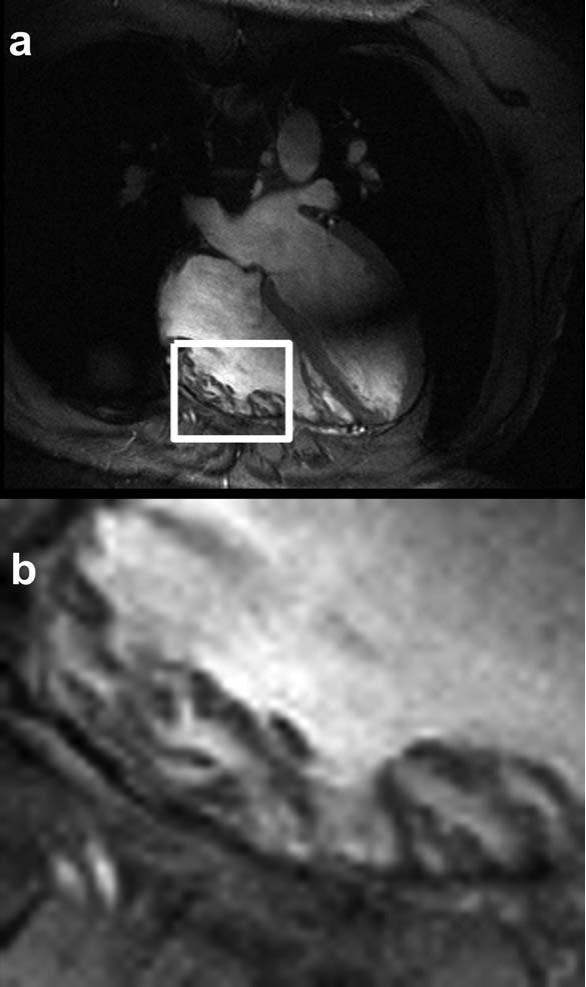
Single frame from a free‐breathing gradient echo cine of the right side of the heart acquired and gated to end expiration using the new diaphragm position estimation. Details of the trabeculation in the right ventricle can be observed, and no observable respiratory artifact is present.

Figure 5 shows one frame of the cine scan; this scan presented no respiratory artefacts and has sharply defined trabeculations on the right ventricle.

## DISCUSSION

A method to estimate diaphragm position using DICO measures from an RF safety supervision system has been presented. The root mean square error is smaller than the window size of a standard diaphragm navigator (1.4 mm compared with 5 mm) 28. A frequency analysis found three dominant sources for the error: The greatest coincides with respiration, as would be expected, as scattering is sensitive to both the liver position and lung‐filling volume, the latter of which may not be linear with diaphragm position. When used in practice as a source for binning or gating, the effect of diaphragm position error will be to shift the bin or gate boundary by a small amount depending on the size of the bin.

The error at the imaging frequency of 0.1 mm is possibly due to the system applying B_0_ eddy current compensation through adjusting the system frequency. As a transmission line coil's scattering is a function of frequency, a change in the measurement frequency will result in a change in scattering. This would be consistent with the authors' observations that this error is linear with respect to the sampled line of k‐space.

In one subject, $\vec{\Gamma }$ was out of its expected range; this can only be because of a substantial change in $S_{0}$, which we believe was caused by the subject or coil moving between the measurement of $S_{0}$ and the validation scan, as a result of subject discomfort. Although the calibration was initially successful, there was a gradual increase in $d_{}^{est}$. We attribute this to the incorrect value of $S_{0}$ initially being compensated by the calibration, but the calculation of $\vec{\Gamma }$ then being unable to correct for drift in ${\vec{v}}_{fwd}$.

The high‐frequency, nonfiltered diaphragm measurement also lends itself to measuring diaphragm velocity, which has enabled the rapid detection of noncyclic events such as coughing. Practically, this can be used to inform the pulse sequence that such an event has occurred, and prospectively hold the progression of encoding steps until the episode is over. Diaphragm velocity could potentially be used to detect end‐inspiration and to separate the data acquired during inspiration from the data during expiration.

The advantage of using RF scattering over self‐navigation methods 4 is that there is no requirement to use a radial encoding strategy or to include navigator readouts 6, which allow the operator to choose both the imaged slice and its encoding freely.

Some readers may be more familiar with noise covariance matrices in the context of a receiver array. Noise covariance, like scattering, is a function of impedance 9, 29. One difference lies in the signal source: Noise covariance are measurements made of small thermally driven fluctuations (Johnson noise) and detected using a temporal correlation in a large set of measurements, whereas scattering measurements use external RF power and require a minimum number of N measurements to measure S(t). Additionally, the diagonal of a noise covariance matrix has no phase information and is only sensitive to changes in tissue conductivity and not permittivity 9.

In conclusion, we have demonstrated a rapid, real‐time, and quantitative method to estimate diaphragm position that uses the scattering of a transmit coil to provide an accurate measure of diaphragm position.

## Supporting information


**Fig. S1. a**: Conductivity map of chosen slice with location of coil elements depicted. **b, c**: Real and imaginary sensitivity of the impedance between coil 3 and coil 2 of a transmission line array centered over the heart. Shown are distinctly different patterns of sensitivity for real and imaginary components.
**Fig. S2**. Real part of the spatial distribution of sensitivity of the impedance between channels i and j. This matrix is symmetric and otherwise distinctly different sensitivity patterns are shown. See color bar in Supporting Figure S1.
**Fig. S3**. Example frequency spectrum of the difference between diaphragm position and scatter scaled to amplitude. Three frequency bands are labeled as respiration, cardiac, and imaging. The spectrum is the Fourier transform of the difference between the scatter measure and the diaphragm position followed by an apodization of 0.15 Hz with a Hanning window, and the amplitude is normalized to the width of the Hann window and frequency bin size. The average heart rate was 59 bpm in this subject. The unlabeled peak at 0 Hz is a constant offset, and the unlabeled peak at 0.6 Hz is expected to be the cross‐modulation of cardiac and respiration frequencies.
