# Supplementary material for: Diaphragm position can be accurately estimated from the scattering of a parallel transmit RF coil at 7 T
Source: Magn Reson Med. 2017 Aug 3;79(4):2164–9. doi: 10.1002/mrm.26866 (PMC5836958; doi:10.1002/mrm.26866)
Supplement: Supplementary file 1 — Fig. S1. a: Conductivity map of chosen slice with location of coil elements depicted. b, c: Real and imaginary sensitivity of the impedance between coil 3 and coil 2 of a transmission line array centered over the heart. Shown are distinctly different patterns of sensitivity for real and imaginary components. Fig. S2. Real part of the spatial distribution of sensitivity of the impedance between channels i and j. This matrix is symmetric and otherwise distinctly different sensitivity patterns are shown. See color bar in Supporting Figure S1. Fig. S3. Example frequency spectrum of the difference between diaphragm position and scatter scaled to amplitude. Three frequency bands are labeled as respiration, cardiac, and imaging. The spectrum is the Fourier transform of the difference between the scatter measure and the diaphragm position followed by an apodization of 0.15 Hz with a Hanning window, and the amplitude is normalized to the width of the Hann window and frequency bin size. The average heart rate was 59 bpm in this subject. The unlabeled peak at 0 Hz is a constant offset, and the unlabeled peak at 0.6 Hz is expected to be the cross‐modulation of cardiac and respiration frequencies. [file MRM-79-2164-s001.docx]

# Supplementary information: Diaphragm position estimation using RF scattering of a parallel transmit coil

## Methods: Electromagnetic simulation of coil sensitivities

A simulation was run of the Transverse Electromagnetic Array (TEM) (1) centred over the heart and used in this paper. The simulation was conducted in Semcad X (Speag, ZMT, Zürich, Switzerland). The simulation used ideal decoupling (that is a single channel was simulated at one time while the others were removed from the simulation). The virtual family member Duke from IT’IS foundation was used in this simulation. The coil was tuned and matched to 50 Ω impedance and the simulation had a resolution of 1 mm isotropic. To calculate the spatial sensitivity of the complex impedance matrix, each coil sensitivity ($\vec{J}_{Li}(v,t)$ in equation 3 of the paper) was calculated as the simulated current field divided by the drive current. The spatial distribution of the sensitivity of the impedance matrix is calculated as:

$$\frac{1}{\sigma\left( \vec{x},t \right)}\vec{J}_{Li}\left( \vec{x},t \right)\cdot\vec{J}_{Lj}\left( \vec{x},t \right)$$

[S1]

## Results: Electromagnetic simulation of coil sensitivities

Plotted in figures S1 and S2 is a transverse slice through the thorax of the spatial distribution of the sensitivity of the impedance matrix in Ωm^-3^. Figure S1 compares real and imaginary components and shows that the real and imaginary components have different sensitivity distributions as is evident in the sign of real and imaginary terms. Figures S2 shows how the real part of all the sensitives vary spatially. Notably there are both positive and negative contributions.

Supporting Figure S1: a) Conductivity map of chosen slice with location of coil elements depicted. b and c) are real and imaginary sensitivity of the impedance between coil 3 and coil 2 of a TEM array centred over the heart. Shown are distinctly different patterns of sensitivity for real and imaginary components.

Supporting Figure S2: Real part of the spatial distribution of sensitivity of the impedance between channels i and j. This matrix is symmetric and otherwise distinctly different sensitivity patterns are shown. See colorbar in figure S1.

Supporting Figure S3: Example frequency spectrum of the difference between diaphragm position and scatter scaled to amplitude, three frequency bands are labelled, respiration, cardiac, and imaging. The spectrum is the Fourier transform of the difference between the scatter measure and diaphragm position followed by an apodization of 0.15 Hz with a Hanning window and the amplitude is normalized to the width of the Hann window and frequency bin size. The average heart rate was 59 beats per minute in this subject. The unlabelled peak at 0 Hz is a constant offset, and the unlabelled peak at 0.6 Hz is expected to be the cross modulation of cardiac and respiration frequencies..
